# Nurses’ Perceptions of the Factors Contributing to the Development of the Love of the Profession: A Qualitative Content Analysis

**DOI:** 10.3390/nursrep11030066

**Published:** 2021-09-09

**Authors:** Mohsen Adib-Hajbaghery, Shahnaz Bolandian-Bafghi, Mitra Zandi

**Affiliations:** 1 Trauma Nursing Research Center, Kashan University of Medical Sciences, Kashan 8715981151, Iran; adib1344@yahoo.com; 2 Faculty of Nursing and Midwifery, Kashan University of Medical Sciences, Kashan 8715981151, Iran; 3 Medical Surgical Nursing Department, School of Nursing and Midwifery, Shahid Beheshti University of Medical Sciences, Tehran 1985717443, Iran; mitra.zandi@yahoo.com

**Keywords:** love of the profession, nursing profession, nursing, qualitative study, content analysis

## Abstract

As a professional value, the love of the profession can significantly affect nurses’ professional practice, behaviors and commitment. Many different factors can affect the love of the profession. The exploration of nurses’ experiences of these factors can provide valuable data for development of the love of the profession. The aim of this study was to explore nurses’ perceptions of the factors contributing to the development of the love of the profession. This qualitative study was conducted in 2020–2021 using the conventional content analysis approach. The participants were thirteen nurses with different organizational positions purposively recruited from different settings in Iran. The data were collected via semi-structured interviews, and were analyzed via the conventional content analysis approach proposed by Graneheim and Lundman. The factors contributing to the development of the love of the profession were categorized into four main categories, namely the public perception of the profession (with three subcategories), educational variables (with two subcategories), the characteristics of the profession (with four subcategories), and nurses’ self-evaluation (with three subcategories). The love of the profession is affected by a wide range of personal, educational, professional and social factors. The manipulation of these factors would help to develop nurses’ and nursing students’ love of the profession, and encourage people to choose nursing as a career.

## 1. Introduction

Nursing is an academic profession in which employees attempt to provide the most appropriate services to the clients through the integration of knowledge and skills [1]. By definition, a profession is a specialized field of study which is based on academic theoretical underpinnings and practical abilities [2]. The professionalization of a field depends on several criteria, including the public need for the services of that profession, emphasis on values, commitment to quality service delivery to all people, adherence to standards, and autonomy [3]. Nursing is also considered as a profession due to its characteristics such as autonomy, commitment, expertise, knowledge and skills, and its value system [4]. The most important characteristic of nursing which contributes to its professionalization is the attention to professional values [5]. 

The love of the profession (LOP) is one of the values of the nursing profession with significant effects on the profession and the provision of professional services [6]. LOP has been defined variously. For instance, Rivero and Erdmann defined LOP in nursing as a sense of interest, concern, respect, understanding and responsibility towards others [7]. Similarly, Rykkje et al. defined LOP as compassion, charity and human love [8]. Some scholars also equated LOP with work engagement and a positive mental approach towards the profession characterized by senses of vigor, dedication and absorption [9]. Vigor is in turn characterized by high levels of energy and resilience at work, while dedication refers to inspiration and the worthiness of the profession, and absorption is the degree to which the profession is pleasant for its employees [10].

LOP is associated with different outcomes. For example, it helps nurses focus on the positive aspects of work, such as pleasure, reward and autonomy, instead of focusing on the negative aspects of work, such as difficult work conditions. In other words, nurses who love their profession mostly see the positive aspects of their profession [11], and hence spend most of their physical, emotional and cognitive energy on work [12]. Moreover, LOP protects nurses against the negative effects of the dominant organizational climate and promotes their long-term professional commitment [13]. Contrarily, a lack of LOP and professional attachment in nurses distances them from their professional roles [14], and thereby causes them to confine themselves to the simple performance of their routine tasks without paying serious attention to human values at work [8]. Nurses with limited LOP are also more likely to experience job burnout [15] and leave their profession [16,17]. Nonetheless, studies have shown low to moderate levels of LOP among nurses [18,19]. Presently in Iran, entering university and the choice of discipline is not based on individual interest, so Iranian students are forced to choose a discipline according to the acquired score in a university entrance exam [20]. Accordingly, a large number of nurses enter without the necessary enthusiasm in the nursing profession. In an earlier study, Adib Haj Bagheri showed that only 60% of students had real interest before choosing their discipline [21]. 

Although nursing textbooks emphasize care based on the nursing process, in clinical education, Iranian students are generally trained in a traditional and task-oriented manner, and are mainly prepared to carry out physicians’ orders [22]. Therefore, nursing is physician-based, and a pivot routine is one of the most important characteristics of Iranian nurses [23]. The reasons mentioned above generally demotivate most nurses and make them less interested in their profession. 

Many different factors can affect LOP. For example, a quantitative study showed that job resources, including physical, social and organizational aspects, significantly affect nurses’ professional engagement [24]. A discourse analysis study in Spain also revealed that work–family conflicts, a sense of justice at work, career advancement opportunities, rewards, and education about communication skills can affect nurses’ professional engagement [25].

Most of the previous studies into the factors contributing to LOP in Iran were conducted using quantitative designs, and hence did not provide detailed data in this area [26,27,28]. Moreover, the findings of the studies conducted in this area in other countries cannot easily be generalized to nurses in Iran. In addition, some previous studies were on concepts similar to LOP, such as professional interest, attachment and engagement. Therefore, there are limited data on the factors contributing to LOP in Iran. The present study was conducted in order to narrow this knowledge gap. 

### Aim of the Study

The aim of the study was to explore nurses’ perceptions of the factors contributing to the development of LOP.

## 2. Methods

This qualitative study was conducted in 2020–2021 using the conventional content analysis approach. This approach helps to describe experiences and identify, code, summarize and categorize the manifest and latent contents in experiences [29]. The COREQ checklist was used to prepare this article and report the study.

### 2.1. Participants

The participants were Iranian nurses and nursing managers who were famous among their colleagues for having a deep LOP. They were purposively recruited to the study with maximum variation from different healthcare settings in five provinces of Iran. Having explained the objectives of the research to the nurse managers, they were asked to introduce those nurses who are enthusiastic and love their profession. They introduced such nurses to the researcher with respect to characteristics they believed are the key to choose: a sense of responsibility, high performance, proper behavior, resilience, passion and a sense of initiation. The participants were also asked to introduce someone they knew who loved the profession. The inclusion criteria were a desire to attend the study, clinical work experience of at least two years, popularity as a nurse with deep LOP among colleagues, the ability to share LOP-related experiences, having a good way with words, and being able to share their experiences in detail. For this purpose, nurses who were witty with the ability to express themselves properly were meant be included in the study.

Before the researcher started the main study, the interview guide was pilot-tested on two nurses to ensure the consistency of the questions’ meanings, and that the questions were clear and comprehensible. These two nurses did not participate in the main study, and their interviews were discarded. 

### 2.2. Data Collection

The data were collected through in-depth semi-structured interviews held by the second author. The interviews were started using general questions in order to gain the participants’ trust and establish a healthy relationship with them. Initially, the participants were asked to describe one of their working days, and then the following questions were used: “Why did you enter nursing?” “May you please explain about the first time you felt that you are interested in nursing?” “May you provide examples of your experiences which show the factors affecting your LOP development?” Moreover, the participants were encouraged to provide clearer explanations about their experiences through asking probing questions such as “May you please provide more explanations?” “What happened that…?” The type of questions used in each interview depended on the intended participant’s answers to the main interview questions. The time and the place of the interviews were arranged based on the participants’ preferences. All of the participants preferred the lounge in their workplace as the place for interviews. The duration of the interviews was 30–90 min. The interviews were audio-recorded using an MP3 recorder. The data collection was kept on until data saturation, i.e., when no new conceptual codes were acquired from the interviews and all of the categories extracted from the data were adequately developed [30]. A second interview was conducted with some of the participants if the researcher encountered any ambiguities or questions while reading and analyzing the interviews. In this way, participants number 1, 2 and 13 were interviewed twice. Saturation was achieved after sixteen interviews with thirteen participants.

### 2.3. Data Analysis

The conventional content analysis approach proposed by Graneheim and Lundman [29] was used for the data analysis. The unit of analysis was considered to be each whole interview. Accordingly, each interview was immediately transcribed word by word in the Microsoft Office word and its transcript was frequently read for immersion in the data. Then, words and sentences related to the study aim were determined and coded. The generated codes were grouped into subcategories according to their similarities. Meanwhile, the subcategories were compared and grouped into larger categories. MAXQDA software 10 (VERBI Software GmbH, Berlin, Germany)was used for the data management.

### 2.4. Trustworthiness

Trustworthiness was ensured using the credibility, dependability, confirmability and transferability criteria. Credibility was established using member checking, prolonged engagement with the study, immersion in the data, and sampling with maximum variation. Dependability was also ensured through peer checking. During the peer checking, two qualitative researchers external to the study assessed and approved the accuracy of the data analysis. Transferability was also ensured by providing detailed descriptions about the data collection, data analysis and findings in order to provide others with the opportunity to link the findings with their experiences. 

### 2.5. Ethical Considerations

The Ethics Committee of Kashan University of Medical Sciences, Kashan, Iran, approved the study (code: IR.KAUMS.NUHEPM.REC.1398.058, issued on 21 December 2019). Participation in the study was voluntary, and the participants could withdraw from the study at will. The aim and the methods of the study were explained to the participants, and their verbal and written informed consents were obtained. Before the interview, with the permission of the participant, the mp3 device was turned on and the recording was started. Prior to that, the participant was assured that his/her audio file would not be available to anyone other than the researcher, his/her words would be used only for study, and the results would be given without mentioning the name and only mentioning a number. The data were managed confidentially, and the findings were reported honestly. Moreover, the participants were ensured that their data would be used solely for the purpose of the present study. All of the audio files of the participants, as well as texts transcribed in Microsoft office word, were written to a DVD and stored in a secure and locked cabinet in the researcher’s office, and will eventually be destroyed.

## 3. Findings

Five male and eight female nurses participated in the present study. Their age and work experience were 30–59 and 7–30 years, respectively. Three of the participants held master’s degrees, and the remaining ten participants held bachelor’s degrees in nursing. Five participants were hospital nurses, two were head nurses, one was a hospital supervisor, and the remaining five were top nursing managers at the local or national level. Three participants had the experience of teaching to nursing students. Table 1 shows the participants’ characteristics.

During the data analysis, 143 primary codes were generated regarding the nurses’ perceptions of the factors contributing to the development of LOP. These codes were categorized into twelve subcategories and the four main categories of the public perception of the profession, the structure of the educational variables, the characteristics of the profession, and the nurses’ self-evaluation (Table 2).

### 3.1. Public Perception of the Profession

Many social factors can affect LOP among nurses. The experiences of some of the participants showed that before entering the profession, they had been affected by social factors such as public respect for nurses, their teachers’ and significant others’ opinions, and nurses’ practice at war. These factors had drawn their interest in nursing. The subcategories of this category were a positive public attitude towards nursing, teachers’ and significant others’ opinions, and inspiration from nurses’ attendance at war. 

#### 3.1.1. Positive Public Attitude towards Nursing

The experiences of six of the participants showed that they had become interested in nursing due to positive media advertisements, public respect for nurses, and people’s gifts to nurses (pp. 1, 3, 5, 7, 11, 13).

*Several years ago, there was a TV show featuring nurses. In some episodes, it showed nurses who visited and provided care to patients. I saw that nurses’ work positively affected patients’ recovery. That show created an interest in nursing in me* (p. 7).

Public respect for nurses and the public admiration of their work had also affected the participants’ engagement and interest in nursing.

*One thing which created interest in nursing in me was people’s respect for me as a nurse. One of my brothers serves in military, the other is a teacher, and I am a nurse. Public respect for me is much greater than the respect for my two brothers* (p. 3).

#### 3.1.2. Teachers’ and Significant Others’ Opinions

The participants’ experiences showed that before entering the nursing profession, the opinions of their teachers, friends, relatives and peers had significantly affected their interest in nursing (pp. 2, 4, 5, 7, 8, 11, 12, 13).

*Teachers had significant effects on my interest in nursing* (p. 7).

*Mrs. X and I were classmates. Her entrance into nursing created an interest in nursing in me* (p. 12).

#### 3.1.3. Inspiration from Nurses’ Attendance at War

Four of the participants reported that they had become interested in nursing due to witnessing nurses’ practice and their help for victims in the Iraq–Iran war. They had noticed that nurses played a significant role in saving the lives of the veterans who fought for the country, and thereby became interested in nursing (pp. 1, 5, 6, 12).

*I went to the war when I was in the fourth year of my high school. There, I witnessed nursing for the first time. It positively affected me. It was a very good experience. Saving the lives of people who were the best people in that period by nurses who were also among the bests was very good. My interest in nursing originates from that period* (p. 5).

### 3.2. Structure of the Educational System 

The second main category of the factors affecting nurses’ LOP was the structure of the educational system of nursing. The experiences of some of the participants showed that despite entering into the nursing profession without serious interest in it, the characteristics of the nursing courses and the behaviors of the nursing instructors had attracted and increased their interest in nursing. The two subcategories of this category were the characteristics of nursing courses and the characteristics of nursing instructors.

#### 3.2.1. Characteristics of Nursing Courses

Six participants noted that the characteristics of nursing courses, such as their applicability in daily life and professional life, as well as their positive experiences during nursing education improved their motivation for learning and positively affected their LOP (pp. 2, 4, 8, 9, 12, 13).

*I liked courses such as medical-surgical nursing. In these courses, we learned about the symptoms of diseases, nursing diagnoses, and nursing care measures, all of which were interesting to me. Educational materials were beneficial both for my job and my life and attracted my interest in nursing* (p. 4).

#### 3.2.2. Characteristics of Nursing Instructors

The experiences of five of the participants showed that their LOP, professional interest and job motivation had greatly been affected by the characteristics of their instructors, such as their knowledgeability, accountability and character (pp. 2, 4, 9, 10, 11).

*We had a good instructor with great professional knowledge. When he explained something for us, medical students came to listen and learn. I told to myself that medical students come to learn from a nurse. It was very pleasant for me and positively affected my interest in nursing. I liked that instructor very much* (p. 2).

*We had good instructors with active engagement in quality teaching to us. They greatly respected me because I was a good student* (p. 4).

### 3.3. Characteristics of the Profession

The third main category of the factors affecting LOP was the characteristics of the profession. According to the participants, the nursing profession has some unique characteristics which positively affect nurses’ LOP. These characteristics were the usefulness of nursing, the diversity of roles, the special body of knowledge, and the holiness of nursing.

#### 3.3.1. Usefulness of Nursing

The usefulness of nursing was one of its main characteristics which positively affected the participants’ LOP and motivated them to enter nursing. The participants noted that nursing is an applied profession in which nurses attempt to help others, reduce their pain and suffering, and fulfill their needs (pp. 1, 2, 3, 4, 5, 6, 7, 8, 9, 10, 11, 12, 13).

*In the first semester, I failed many courses because I had no interest in nursing. I experienced depression. In the second semester, my father experienced a myocardial infarction followed by cardiac arrest at the age of 47–48 years. He was successfully resuscitated using defibrillation. As I was at his bedside, I witnessed how nursing staff worked [to resuscitate my father]. The resuscitators of my father were nurses rather than doctors. Nurses’ practice significantly changed my approach to nursing and attracted my interest in nursing* (p. 8).

#### 3.3.2. Diversity of Roles

The diversity of the available roles and positions for nurses in different settings—such as healthcare settings, educational settings and ministries—positively affected the participants’ interest in nursing and their LOP. The participants noted that nurses can play a role as staff nurse, head nurse, hospital supervisor, hospital nursing manager, deputy minister, researchers, and instructors. The diversity of the available roles for nurses provides them with adequate career advancement opportunities, and thereby has positive effects on their professional interest and LOP (pp. 5, 6, 8, 10, 11).

*Role diversity makes me interested in nursing very much. Nurses can act as a drug administrator, hospital supervisor, manager, faculty member, and deputy minister. The route is open for you and you can become even a deputy minister* (p. 5).

#### 3.3.3. Special Body of Knowledge

According to the participants, the special body of knowledge of nursing, up-to-date knowledge of the profession, the integration of knowledge and practice, and the liveliness of the profession have significant effects on nurses’ professional interest and LOP. In fact, when nurses understand that their profession is based on a firm body of knowledge, they will have greater interest in it (pp. 1, 2, 4, 5, 9, 11). As one of the participants who had changed her field of study from the operating room field to the nursing field noted:

*A nurse needs to study, be up-to-date, and learn many things. In my opinion, nursing is associated with learning more things in comparison with the operating room field. In the operating room field, you need to know a lot about human anatomy and work as an assistant to physicians. However, in nursing, you need to have much more information. I love nursing for this reason* (p. 4).

#### 3.3.4. Holiness of Nursing

According to some of the participants, the nursing is a profession which is based on helping others, and hence can be considered a non-materialistic and holy profession (pp. 1, 8, 9, 10, 12).

*Nursing has spiritual value for me. It is a holy profession which cannot be related to materialistic values. Therefore, I like it very much* (p. 8).

### 3.4. Nurses’ Self-Evaluation

The fourth main category of the factors affecting nurses’ LOP was their self-evaluation. The participants noted that their self-evaluation of their practical skills, professional knowledge and job satisfaction had contributed to the development of their LOP. The three subcategories of this category were practical skills, professional knowledge, and perceived job satisfaction. 

#### 3.4.1. Practical Skills

Technical and practical nursing skills give nurses a sense of satisfaction and promote their job motivation and LOP. Examples of these skills include venipuncture, cardiopulmonary resuscitation, and endotracheal intubation (pp. 2, 3, 6, 8, 9).

*When I started my work as a nurse, my interest in nursing significantly increased. For example, I felt satisfaction and pleasure when I performed a successful CPR or established an intravenous line for a neonate* (p. 9).

#### 3.4.2. Professional Knowledge

Professional knowledge in areas such as electrocardiography, medication side effects, the diagnosis of disease, and nursing diagnoses provided the participants with a positive self-evaluation and resulted in their greater LOP. In fact, nurses feel greater LOP and professional interest when they feel that learning in their profession is ongoing (pp. 1, 2, 5, 8, 6, 11, 12).

*I learned many things when I started nursing practice. For example, I learned many things about diseases, medications, and medication side effects. Such learning increased my interest in nursing* (p. 11).

#### 3.4.3. Job Satisfaction

All of the participants noted that the positive effects of their interventions for patients improved their job satisfaction, increased their professional interest, and promoted their LOP (pp. 1, 2, 3, 4, 5, 6, 7, 8, 9, 10, 11, 12, 13).

*When I see the recovery of patients and the happiness of them and their families, I feel satisfaction and feel that my life has not been fruitless. This is the reason for my love of nursing* (p. 5).

## 4. Discussion

This study explored nurses’ perceptions of the factors contributing to the development of LOP. There were no studies conducted—or at least available—both nationally and internationally in the area of LOP among nurses in spite of its importance. Therefore, it can be claimed that this study is one of the few studies performed in the field of LOP in nurses. The findings came in four main categories, namely public perception of the profession, the structure of the educational system, the characteristics of the profession, and nurses’ self-evaluation.

**Public perception of the profession**: The first main category of the study was the public perception of the profession. The findings showed that some of the participants had become interested in nursing before entering it due to the effects of society on them. A study reported that the acceptable public image of a profession significantly affects the development of professional identity among the workers in that profession [31]. Another study showed that the public image of nursing can affect high school students’ choice of nursing as a career. For example, the negative attitudes of people in the United Kingdom have caused them not to accept nursing as a profession [32]. Similarly, a study in Saudi Arabia showed that choice of nursing as a career is greatly affected by the public image of the profession, such that social norms in that country prevent people from entering the profession [33]. Contrarily, a positive image of nursing in the United States motivates people to enter nursing [34]. The public image of a profession not only affects the attitudes of people outside that profession but also affects the image of people in that profession of themselves and their profession. A study showed that nurses’ image of nursing is affected by the public image of nursing, as well as the amount of public respect and value for the profession [35].

The study findings also revealed that one of the social factors affecting nurses’ LOP was their significant others, relatives, and teachers. In line with this finding, a former study in Iran showed that family, relatives and friends had significant effects on individuals’ willingness to enter nursing [36]. Similarly, a study showed that a main reason contributing to nursing students’ willingness to enter nursing was the effects of their significant others, particularly their family members [37]. Another study also found that for entering nursing, male nurses faced the negative reactions of their male significant others, such as their fathers and friends [38]. 

The study findings also indicated that some of the participants had become interested in nursing due to their experience of attending war zones and witnessing nurses’ practice at war. In line with this finding, previous studies showed that war has been a significant factor in changing the profession of nursing. For example, the Crimean war caused significant changes in nursing in the United States, and turned it from a traditional home-based care delivery task to an advanced profession [39]. People who attend war zones are usually considered to be heroes. Heroes usually have significant roles in boosting others’ morale, promoting ethical practice, and protecting people against threats [40]. Similarly, nurses who attend war zones to save victims’ lives are considered to be heroes, and become role models for others [41]. Nurses’ courage at war is associated with a positive public image of nursing, and stimulates others’ desire to enter nursing [42]. This fact is not limited to conventional wars between countries; rather, nurses’ practice in the frontline of care delivery in the coronavirus disease 2019 (COVID-19) pandemic has improved the public image of nursing, and has turned them into healthcare heroes [43]. This attitude shift can turn nurses into role models for others, stimulate others’ desire to enter nursing, and increase nurses’ interest in their own profession. 

What makes the current study different from others is our results concerning the impact of mass media on the love for the profession. The findings of this study showed that a number of the participants became interested in the nursing profession after watching a film or series related to this profession, indicating a positive impact on them. However, the results of previous studies show that Iranian nurses are dissatisfied with the stereotypical images of the nursing career in the media, and in this regard, the media has had a negative impact on their interest [44,45].

**The structure of the educational system**: The second main category of the study was the structure of the educational system of nursing. The findings showed that the educational system of nursing and its characteristics—such as the practice of nursing instructors—can affect nurses’ LOP. A former study in Iran also showed that nursing instructors’ behaviors had significant effects on nursing students’ professional interest [21]. Another study showed that nursing instructors’ behaviors, practice and communication skills can affect the learning climate, students’ motivation for learning, and their self-confidence [46]. Contrarily, an inappropriate learning climate, an ineffective curriculum, and limited support from instructors can result in burnout, boredom and disinterest among nursing students [47]. It is noteworthy that students’ negative attitudes towards the nursing curriculum and educational materials negatively affect their learning in the cognitive, affective and psychomotor domains, and lead to their disinterest in nursing [48].

**The characteristics of the profession**: The third main category of the study was the characteristics of the profession. Some of the participants noted that their LOP originated from the unique characteristics of the nursing profession, including its usefulness, role diversity, special body of knowledge, and holiness. This is in line with the findings of several former studies. For example, a study revealed that professional autonomy, the giving of feedback, professional identity, and professional importance were the significant predictors of professional engagement [49]. Another study also revealed that some professional characteristics, such as autonomy and positive feedback, had a significant positive relationship with nurses’ professional engagement, and encouraged nurses’ motivation for remaining in the profession [50]. Similarly, a study indicated that the characteristics of nursing—such as role diversity, professional skills, professional identity, professional importance and professional autonomy—significantly contributed to the development of professional engagement among nurses [51].

The holiness of nursing was another characteristic of the profession which had affected our participants’ LOP. In line with this finding, nurses and lay people in a former study considered nursing to be a holy activity [52]. Such a consideration can result in greater professional interest among nurses. Similarly, spiritual values and the holiness of the profession—as highlighted during the COVID-19 pandemic—have improved nurses’ resilience and increased their professional interest [53]. These findings imply that nurses’ more positive attitudes towards the characteristics of their profession are associated with greater professional interest and LOP.

**Self-evaluation**: The fourth main category of the factors affecting LOP among nurses was the nurses’ self-evaluation. The findings revealed that the participants’ evaluation of their practical skills, professional knowledge, and satisfaction with their professional activities had affected their LOP. In line with this finding, a study revealed that the self-evaluation of professional abilities significantly affected professional engagement [54]. Another study also showed a significant relationship between clinical self-efficacy and clinical belongingness [55]. Moreover, a study highlighted that a positive attitude towards one’s professional skills and abilities is associated with greater self-esteem and greater self-efficacy [56]. Self-efficacy, in turn, enhances nurses’ job satisfaction and subsequently increases their professional engagement and interest [57].

### Limitations

The limitations in qualitative research may be related to the lack of objectivity between the researcher and the subject. In addition, in qualitative research, the participants may not share all of their experiences on the subject, and may have other considerations, for which the researcher should seek to gain the participants’ trust by communicating effectively and removing this barrier insofar as is possible. Because the researcher interviewed some of the participants twice, this may have caused differences between the content of the interviews conducted among some of the participants. The findings of this work might also be affected by gender, as a large number of female nurses participated in the study. However, this is due to the large number of female nurses in Iran. Among the limitations of the study was the refusal of three nurses to participate in the study due to their fear over affliction by COVID-19, as well as their great workload and fatigue. 

## 5. Conclusions

This study concludes that as an ethical value of the nursing profession, LOP is affected by different personal, educational, professional and social factors, namely the public perception of the profession, the structure of the educational system, the characteristics of the profession, and nurses’ self-evaluation. Therefore, strategies such as improving the public perception of nursing, improving the educational system of nursing, strengthening its curriculum, recruiting more competent instructors, creating a more positive learning environment, promoting nurses’ professional autonomy, and providing them with constructive feedback are recommended in order to increase nursing students’ and nurses’ professional interest and develop their LOP. These strategies can also improve their image of the profession, their job satisfaction, and their job motivation.

## Figures and Tables

**Table 1:** The participants’ demographic characteristics.

No.	Age (Years)	Gender	Work Experience (Years)	Degree	Position	Unit
1	44	Female	18	Bachelor’s	Nurse	Intensive care
2	44	Female	17	Bachelor’s	Supervisor	Nursing office
3	50	Male	26	Bachelor’s	Head nurse	Surgical care
4	41	Female	16	Bachelor’s	Nurse	Surgical care
5	52	Male	30	Master’s	Nurse	University
6	60	Male	30	Master’s	Manager	Nursing trade union
7	32	Female	4	Bachelor’s	Nurse	COVID-19 care
8	40	Male	16	Master’s	Relief staff	Emergency medical services
9	38	Female	13	Bachelor’s	Infection control staff	Accreditation
10	30	Male	7	Bachelor’s	Head nurse	Neurosurgical care
11	38	Female	15	Bachelor’s	Supervisor	Nursing office
12	38	Female	11	Bachelor’s	Nurse	Coronary care
13	46	Female	19	Bachelor’s	Nurse	Dialysis

**Table 2:** Factors contributing to the development of LOP from the perspectives of the nurses.

Main Categories	Subcategories
Public perception of the profession	- Positive public attitude towards nursing
	- Teachers’ and significant others’ opinions
	- Inspiration from nurses’ attendance at war
Structure of the educational system	- Characteristics of nursing courses
	- Characteristics of nursing instructors
The characteristics of the profession	- Usefulness of nursing
	- Diversity of roles
	- Special body of knowledge
	- Holiness of nursing
Self-evaluation	- Practical skills
	- Professional knowledge
	- Job satisfaction

## Data Availability

All of the raw data (the participants’ voice files and the texts of the interviews) are confidential, and cannot be provided to anyone. However, the codes which emerged during the current study are available from the corresponding author upon reasonable request.
